# The Compression Flow as a Measure to Estimate the Brain Connectivity Changes in Resting State fMRI and 18FDG-PET Alzheimer's Disease Connectomes

**DOI:** 10.3389/fncom.2015.00148

**Published:** 2015-12-16

**Authors:** Antonio G. Zippo, Isabella Castiglioni, Virginia M. Borsa, Gabriele E. M. Biella

**Affiliations:** ^1^Institute of Molecular Bioimaging and Physiology, Department of Biomedical Sciences, National Research CouncilSegrate, Italy; ^2^Division of Neuroscience, San Raffaele Scientific InstituteMilan, Italy

**Keywords:** 18FDG-PET, RS-fMRI, Alzheimer's disease, voxelwise functional connectivity, functional integration

## Abstract

The human brain appears organized in compartments characterized by seemingly specific functional purposes on many spatial scales. A complementary functional state binds information from specialized districts to return what is called *integrated information*. These fundamental network dynamics undergoes to severe disarrays in diverse degenerative conditions such as Alzheimer's Diseases (AD). The AD represents a multifarious syndrome characterized by structural, functional, and metabolic landmarks. In particular, in the early stages of AD, adaptive functional modifications of the brain networks mislead initial diagnoses because cognitive abilities may result indiscernible from normal subjects. As a matter of facts, current measures of functional integration fail to catch significant differences among normal, mild cognitive impairment (MCI) and even AD subjects. The aim of this work is to introduce a new topological feature called *Compression Flow* (CF) to finely estimate the extent of the functional integration in the brain networks. The method uses a Monte Carlo-like estimation of the information integration flows returning the compression ratio between the size of the injected information and the size of the condensed information within the network. We analyzed the resting state connectomes of 75 subjects of the Alzheimer's Disease Neuroimaging Initiative 2 (ADNI) repository. Our analyses are focused on the 18FGD-PET and functional MRI (fMRI) acquisitions in several clinical screening conditions. Results indicated that CF effectively discriminate MCI, AD and normal subjects by showing a significant decrease of the functional integration in the AD and MCI brain connectomes. This result did not emerge by using a set of common complex network statistics. Furthermore, CF was best correlated with individual clinical scoring scales. In conclusion, we presented a novel measure to quantify the functional integration that resulted efficient to discriminate different stages of dementia and to track the individual progression of the impairments prospecting a proficient usage in a wide range of pathophysiological and physiological studies as well.

## Introduction

The human brain exhibits an incredibly large repertoire of computations. From the spinal cord and the retina, most of input flows along subcortical regions and to cortices. Stages of data processing come in succession in a parallel and distributed manner that is yet mostly debated. One of the major insights in this thread claims that the human brain appears organized in sections defined by rather specific function in a complex architecture that span several spatial scales from neuronal to regional levels. In a concurrent and fundamental functional state, the human brain combines information from the specialized districts exhibiting what is called *functional information integration* (Tononi et al., 1994).

The level of information integration rises and falls during sleep and wakefulness and in many other physiological and pathological conditions. Since its formulation (Tononi et al., 1994), the information integration has received a plethora of supporting evidences and many methods to measure it have been proposed. The most accepted formulation defines the human brain network in terms of the average shortest path length among all possible node couples, a concept called *characteristic path length* (L) (Watts and Strogatz, 1998). Despite the very intuitive and simple definition, the characteristic path length suffers from many limitations and pitfalls. Two among all, it considers all paths as equiprobable neglecting preferential routes fostered by the topology and, secondary, it averages over all non-infinite paths underestimating the contribution of unconnected nodes.

Functional integration as well as other fundamental network dynamics undergoes to severe disarrays in a number of diverse pathological and degenerative conditions such as epilepsies, Parkinson or Alzheimer's Diseases (AD). In the current view, the AD represents a composite syndrome characterized by structural, functional, and metabolic landmarks (Brier et al., 2014a). Indeed, atrophy and hypometabolism accompanying cascades of intervening degenerations eventually leading to full blown dementia (Chase, 2014). However, clear etiological features at the level of brain connectome still miss. In particular, in the early stages of AD when occur a relevant amyloid accumulation, adaptive functional modifications of the brain networks mislead initial diagnoses because cognitive abilities may result indiscernible from normal subjects (Lim et al., 2014). As a matter of facts, current measures of functional integration fail to catch significant differences among normal, mild cognitive impairment (MCI) and even AD subjects.

The aim of this work is to introduce a new topological feature called *Compression Flow* (CF) able to finely estimate the extent of the functional integration in the brain networks. The method produces a set of random walks driven by network centrality criteria that would represent inferred routes of information the information processing flow. The estimation of the information integration is obtained as the compression ratio between the size of the injected information generating the random walks and the size of the condensed information within the network.

To verify the efficacy of proposed method, we compared and analyzed CF and L values on two models of brain-like networks where we simulated a sort of structural degeneracy by progressively removing edges by chance.

Subsequently, we analyzed the functional connectomes of 75 subjects of the Alzheimer's Disease Neuroimaging Initiative (ADNI) repository (http://adni.loni.usc.edu/). Since recent specific literature suggests that [18F]2-fluoro-2-deoxyglucose Positron Emission Tomography (18FDG-PET) and the resting-state functional magnetic resonance imaging (fMRI) carry out consistent and complementary characterization of AD and its prodromal stages, our analyses are focused on both the 18FGD-PET and the fMRI acquisitions in the four different conditions of normal (CR), early MCI (EMCI), late MCI (LMCI), and AD.

Results indicated that CF can effectively discriminate MCI, AD, and normal subjects by showing a significant decrease of the functional integration in the AD and MCI brain connectomes. Furthermore, AD connectomes showed lower functional integration capability in comparison to MCI connectomes accordingly to the phenomenological severity of the disease progression. More importantly, CF could track the disease progression within each of three pathological classes (EMCI, LMCI, AD), a property that we did not observe with other standard complex network statistics. In conclusion, we presented an *in silico* biomarker to quantify the functional integration that resulted efficient to discriminate different stages of dementia and to differentiate the follow-up groups in AD, EMCI, and LMCI and would be proficiently applied into a wider range of pathophysiological and physiological studies as well.

## Materials and methods

### Subject data

Data used in the preparation of this article were obtained from the ADNI database (adni.loni.usc.edu). The ADNI was launched in 2003 as a public-private partnership, led by Principal Investigator Michael W. Weiner, MD. The primary goal of ADNI has been to test whether serial magnetic resonance imaging (MRI), positron emission tomography (PET), other biological markers, and clinical and neuropsychological assessment can be combined to measure the progression of MCI and early Alzheimer's disease (AD). For up-to-date information, see www.adni-info.org. We selected participants subject to the protocol ADNI2 that involves the scanning modalities of the functional neuroimaging such as the 18FDG-PET and the functional Magnetic Resonance Imaging (fMRI). From the second version of the ADNI (ADNI2), we selected 75 subjects by applying the following criteria: (i) the subject had at least two functional acquisitions (18FDG-PET or fMRI) in time; (ii) each functional acquisition was related to an anatomical acquisition within a maximum period of 2 months. After the above selection, we had a total of 75 subjects, 24 Normal, 18 EMCI, 16 LMCI, and 17 AD.

### Longitudinal analysis

According to the ADNI acquisition guidelines, due to risks associated to the contrast medium, subjects can receive a 18FDG-PET acquisition once a couple of year with a tolerance of few weeks. Instead, fMRI acquisition can be achieved up to twice a year again with few weeks of tolerance. At the time of our analyses, ADNI dataset provided subjects (irrespective to the initial screening assessment) with up to two 18FDG-PET sessions (year 0, year 2) and up to five fMRI sessions (months 0, month 6, month 12, month 18, month 24).

### Signal processing

**Figure 1:**
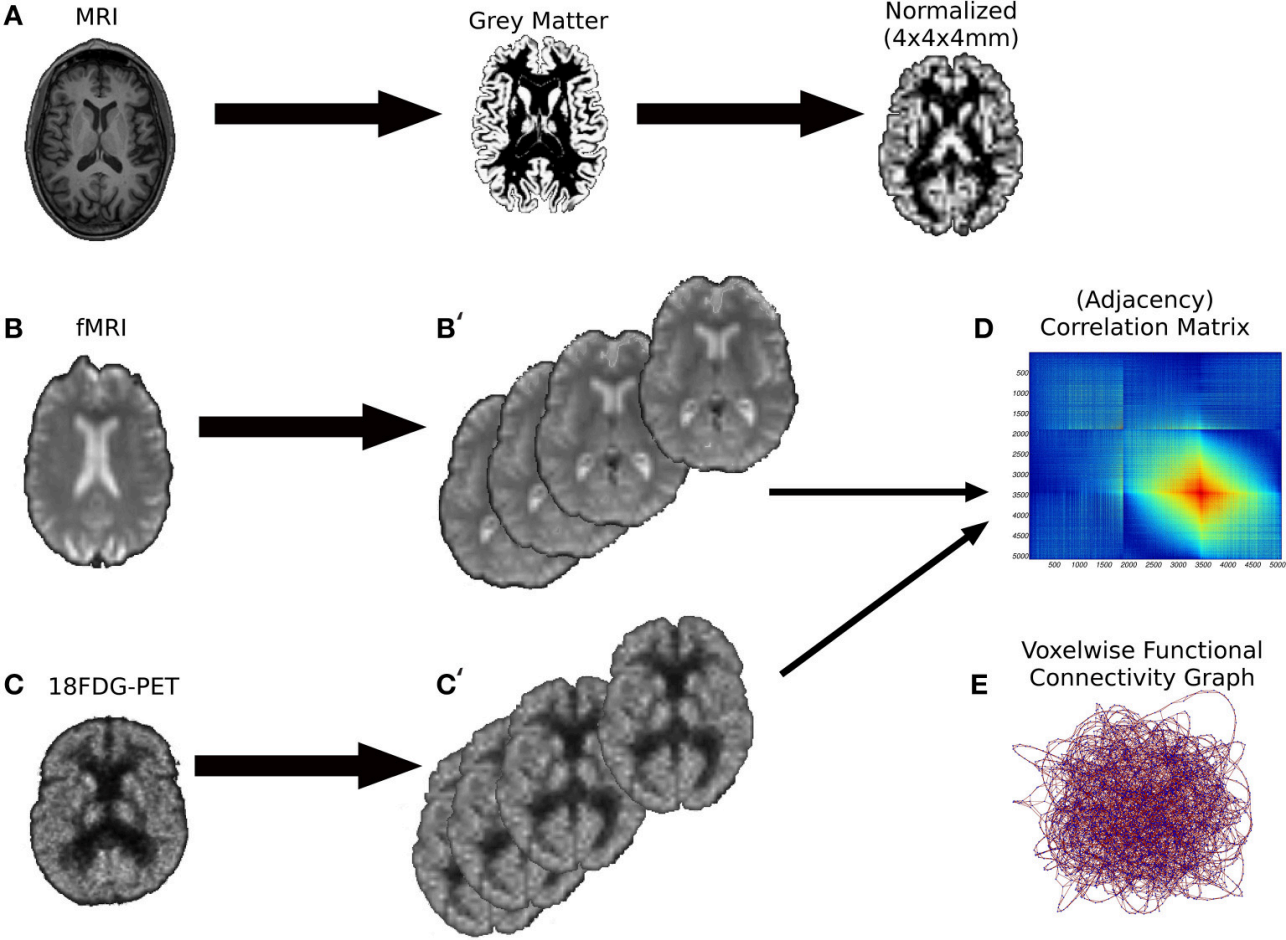
**The preprocessing pipeline of the images**. **(A)** First we performed a brain segmentation on the MRI anatomical volumes keeping the gray matter probabilistic map. Subsequently, the gray maps were spatially normalized onto a MNI space of 4 × 4 × 4 mm voxels. **(B,B')** Resting-state fMRI volumes underwent to several processing phases (alignment and unwarping, coregistration with anatomical volumes, spatial normalization) to extract the Pearson's correlation matrices **(D)**, the counterpart of weighted graphs **(E)**. A similar preprocessing procedure was performed for 18FDG-PET volumes **(C,C')**.

Data were preprocessed and analyzed using SPM12 (Statistical Parametric Mapping, http://www.fil.ion.ucl.ac.uk/spm/software/spm12, Wellcome Department of Cognitive Neurology, London, UK). Prior to analysis, all images for four sessions underwent a series of preprocessing steps according to a stable pipeline (see Figure 1).

Anatomical volumes were segmented in order to extract the gray matter probabilistic map also saving the bias corrected map for the subsequent gray matter image normalization. Since volumes were processed in the MNI space, we selected the “forward deformation” as deformation field. We used a sampling distance of 1 mm with the respect to the smallest dimension of voxel that was of 1.05 mm. All other parameters were set to the default values of the “Segment” module of SPM12. After segmentation, we normalized the gray matter probabilistic map by using the deformation field with a uniform voxel size of 4 mm. Finally, in this gray matter space, we selected voxels with probability equal to 1 which constituted the gray matter mask for the functional volumes.

Volumes obtained from the fMRI (140 for each recording session, cutting off possible longer acquisitions, with typical voxel size 3.31 × 3.31 × 3.31 mm) essentially underwent to three preprocessing steps. First, all volumes passed through a realignment phase to correct spatial effects primarily due to head movements during the scanning. Subsequently, an unwarping stage reduces unwanted variances caused by the effects of the magnetic field inhomogeneities (Andersson et al., 2001). Realignment used a separation distance of 1 mm and the estimation procedure required the best available quality (“Quality” equal to 1). Then, we co-registered the obtained images by using the original anatomical image as reference and the mean of realigned/unwarped images as source (Andersson et al., 2001). At last, we normalized the co-registered images by means of the anatomical deformation field by using a voxel size of 4 mm.

Although the better achievable resolution was 3.31 mm, after a preliminary analysis, we chosen the resolution of 4 mm because 3.31 mm voxels typically produces more than 10.000 gray matter voxels that made the subsequent computation of functional graph statistics unfeasible for our computational setups (see Discussion for considerations).

All 18FDG-PET volumes (six for each recording session) were acquired with a typical voxel size of 2 × 2 × 2 mm and underwent to similar preprocessing pipeline except for the estimation procedure of realignment that used a smoothness of 7 mm for the Gaussian kernel. In addition, PET images were registered to the mean of the images instead of to the first images as in the fMRI processing.

### Functional connectivity estimation

Once the gray matter voxels have been extracted from the functional volumes, we reshaped the 4D signals into bi-dimensional matrices where the *m* rows represented the time slots and the *n* columns represented the voxel IDs. For each acquisition, the estimation of the Pearson correlation coefficient returned a symmetric square matrix $M={\left([0,1]\subseteq \mathbb{R}\right)}^{n\times n}$ where the (*i, j*) element indicated the strength of the functional connection between nodes *i* and *j*. Since we computed the statistical significance of each extracted correlation coefficient, we kept only those that had a *p*-value smaller than 0.05. The matrix M was the adjacency matrix of the resulting graph *G*, the functional connectome of interest. All graphs were maintained in their weighted form. We analyzed the functional connectivity graphs with a set of common network statistics preferring to keep graphs in their original weighted form avoiding thresholding techniques which produce loss of information, complicate analyses because of the introduction of the threshold parameter and are used when the network statistics of interest cannot be applied to weighted graphs (Rubinov and Sporns, 2011).

### Complex network statistics

For the analysis of these graphs, we introduced a set of common statistics from the Complex Network Theory able to estimate the network extent to integrate and segregate information and many other relevant network features (see Table 1). Functional segregation and integration in networks can be measured by two statistics: the clustering coefficient (C) and the characteristic path length (L). The former measures how close the neighbors of a node are to being a clique. The latter estimates the average shortest path length in the graph, i.e., how much the nodes are accessible. Computationally, analyses on the extracted functional brain networks were performed in Matlab by the Brain Connectivity Toolbox (BCT; Rubinov and Sporns, 2010) and by other routines developed in our lab.

**Table 1:** **The set of subjects selected from the ADNI2 repository**.

**ADNI ID**	**Diagnosis**	**Modalities**	**Age**	**FAQ**	**GDSCALE**	**CDR**	**MMSE**
002_S_4213	Normal	rs-fMRI, 18FDG-PET	78.09	0	1	0	28
002_S_4237	EMCI	rs-fMRI, 18FDG-PET	80.98	0	1	0.5	29
002_S_4270	Normal	rs-fMRI	74.73	0	3	0	28
002_S_4473	EMCI	rs-fMRI	74.91	0	2	0.5	27
002_S_4654	LMCI	rs-fMRI, 18FDG-PET	75.47	3	0	0.5	29
002_S_4219	LMCI	rs-fMRI	79.50	0	1	0.5	30
006_S_4150	Normal	rs-fMRI	73.97	0	0	0	28
006_S_4153	AD	rs-fMRI, 18FDG-PET	79.39	4	1	0.5	22
006_S_4192	AD	rs-fMRI, 18FDG-PET	82.38	4	2	0.5	19
006_S_4515	LMCI	18FDG-PET	74.72	8	2	0.5	26
006_S_4867	AD	18FDG-PET	74.51	24	2	1	23
006_S_5153	AD	18FDG-PET	79.06	1	3	0	25
010_S_4442	Normal	18FDG-PET	74.34	0	0	0	30
011_S_4120	Normal	18FDG-PET	81.87	0	0	0	30
011_S_4222	Normal	18FDG-PET	82.38	0	1	0	30
018_S_2180	EMCI	rs-fMRI	77.82	2	2	0.5	30
018_S_4313	Normal	rs-fMRI, 18FDG-PET	77.15	0	0	0	25
018_S_4399	Normal	rs-fMRI, 18FDG-PET	78.00	0	0	0	28
018_S_4733	AD	rs-fMRI	75.37	26	1	1	26
018_S_4809	EMCI	rs-fMRI	78.33	0	1	0.5	24
018_S_4868	EMCI	rs-fMRI	77.21	0	0	0.5	27
018_S_4889	LMCI	rs-fMRI	75.69	2	1	0.5	28
019_S_4252	AD	rs-fMRI	86.58	27	1	1	22
019_S_4285	EMCI	rs-fMRI	74.08	0	2	0.5	29
019_S_4477	AD	rs-fMRI	82.21	16	0	1	21
019_S_4548	LMCI	rs-fMRI, 18FDG-PET	84.85	0	2	0.5	26
019_S_4549	AD	rs-fMRI	79.11	3	1	0.5	21
019_S_4835	Normal	rs-fMRI	79.39	0	0	0	26
019_S_5012	AD	rs-fMRI	76.36	6	3	0.5	25
024_S_4158	Normal	18FDG-PET	84.41	0	2	0	30
024_S_4392	EMCI	18FDG-PET	82.60	3	2	0.5	29
033_S_4179	Normal	18FDG-PET	83.04	0	1	0	30
036_S_4430	LMCI	18FDG-PET	80.07	4	0	0.5	24
037_S_4071	Normal	18FDG-PET	84.67	3	0	0	24
037_S_4302	LMCI	18FDG-PET	76.14	3	2	0.5	25
053_S_4557	EMCI	rs-fMRI, 18FDG-PET	83.42	4	6	0.5	27
073_S_0311	Normal	18FDG-PET	78.19	0	0	0	30
073_S_0746	LMCI	18FDG-PET	73.91	2	2	0.5	30
073_S_4300	LMCI	18FDG-PET	80.61	0	1	0.5	26
073_S_4382	Normal	18FDG-PET	75.92	0	0	0	28
116_S_4043	Normal	18FDG-PET	82.14	0	0	0	29
130_S_2373	EMCI	rs-fMRI	79.13	1	1	0.5	29
130_S_2403	EMCI	rs-fMRI	79.30	9	2	0.5	29
130_S_4250	LMCI	rs-fMRI, 18FDG-PET	78.58	10	2	0.5	29
130_S_4294	LMCI	rs-fMRI, 18FDG-PET	75.42	0	1	0.5	28
130_S_4343	Normal	rs-fMRI, 18FDG-PET	79.72	0	0	0	30
130_S_4352	Normal	rs-fMRI, 18FDG-PET	83.70	0	0	0	29
130_S_4415	EMCI	rs-fMRI, 18FDG-PET	75.28	11	1	0.5	28
130_S_4417	EMCI	rs-fMRI	74.52	0	3	0.5	30
130_S_4542	LMCI	rs-fMRI	79.36	20	4	0.5	25
130_S_4589	AD	rs-fMRI	75.20	15	1	1	26
130_S_4605	LMCI	rs-fMRI	84.77	7	2	0.5	29
130_S_4660	AD	rs-fMRI, 18FDG-PET	77.30	8	1	0.5	24
130_S_4730	AD	rs-fMRI	81.17	16	1	1	21
130_S_4883	EMCI	rs-fMRI	76.18	3	3	0.5	29
130_S_4925	LMCI	rs-fMRI	75.10	8	1	0.5	27
130_S_4971	AD	rs-fMRI	76.54	18	2	1	21
130_S_4990	AD	rs-fMRI	75.19	19	1	1	25
130_S_5142	AD	rs-fMRI	76.42	0	1	0	30
130_S_5175	EMCI	rs-fMRI	79.58	0	0	0	30
135_S_4281	EMCI	18FDG-PET	77.56	0	1	0.5	27
135_S_4406	LMCI	18FDG-PET	78.96	3	3	0.5	29
135_S_4566	Normal	18FDG-PET	83.52	0	0	0	29
136_S_0186	Normal	rs-fMRI	80.53	0	2	0	27
136_S_4433	Normal	rs-fMRI	76.92	0	0	0	30
137_S_4211	AD	18FDG-PET	81.01	3	3	1	22
137_S_4258	AD	18FDG-PET	75.95	10	1	0.5	24
137_S_4299	EMCI	18FDG-PET	76.93	1	3	0.5	25
137_S_4331	EMCI	18FDG-PET	75.43	2	2	0.5	29
137_S_4466	Normal	18FDG-PET	79.85	0	2	0	30
137_S_4482	Normal	18FDG-PET	77.31	0	0	0	28
137_S_4536	EMCI	18FDG-PET	77.98	0	1	0.5	28
141_S_1004	LMCI	18FDG-PET	74.08	20	4	0.5	27
941_S_4100	Normal	18FDG-PET	78.62	0	1	0	28
941_S_4365	Normal	18FDG-PET	80.36	0	0	0	29

*The first column represents the univocal ID assigned at each subject. The second column indicates the clinical diagnosis at the time of the initial screening. The third column shows the modalities of acquisition of the functional volumes. The fourth column represents the age at the initial screening. The total score of the Functional Assessment Questionnaire (FAQ) test is reported at column fifth. The total score of the Geriatric Depression Scale (GDSCALE) test is reported at column sixth. The global score of the Clinical Dementia Rating (CDR) test is indicated at column seventh and the total score of the Mini-Mental State Examination (MMSE) test is showed in the last column*.

The functional graphs obtained by our analysis were further characterized to study the information workflow. To this aim, we estimated the network centrality with the notions of betweenness and eigenvector centrality (Gould, 1967; Freeman, 1979). Because it can be interpreted as a measure of the importance of a node within the network, the distribution of node centrality highlights how the information workflow is distributed among nodes.

We further studied the modularity and the community structure of our graphs but preliminary analyses performed with the two most used community detection algorithms (Newman, 2006; Blondel et al., 2008) reported modularity values close to zero nullifying the validity of the computed partitions. The problem was due to the well-known tendency of such algorithms to prefer clusters of big size. Therefore, we decided to tweak the multi-resolution parameter in order to force the Louvain algorithm to produce modules of smaller dimension (gamma = 1.15 in all analyses). By using this trick, we obtained better values of modularity and we proceeded with inferences.

Ultimately, we analyzed networks that evolved in time potentially dropping and recruiting nodes and connections that come from different experimental conditions. Such a methodology requires the discussion of potential issues. We performed network analyses on the original weighted version of graphs instead of using binarization techniques for several reasons. First, unconnected nodes can be rare but could occur especially after adjacency matrices binarization. Network comparisons with different number of nodes require hard statistical analyses and to discard incompatible networks (for instance those with too few nodes or with more than one strongly connected component) from observations. Second, graph thresholding produces inevitably loss of information. Hence the selective removal of portions of weights almost surely will reduce the power of the consequent network statistics. Third, when thresholding graphs, it is necessary to repeat computations for a discrete range of subjective values thus producing a considerable increase of the overall computational complexity (see Discussion in the main manuscript). Last, all network statistics that we needed for our analyses have a weighted counterpart (Rubinov and Sporns, 2011).

### Compression flow

The aim of this work was to propose a new measure of information integration able to catch dynamical features of the brain network information flow. Despite the large amount of proposed methods, an accurate metrics able to track substantial changes in the in information integration capability is still missing.

We proposed a complementary measure of information integration inspired by fundamental theoretical tenets:

- The human brain processes information by means of a crucial step, the *functional integration*. In a computational perspective information integration is a data compression operation where raw input data from senses produce or recollect high-order cognitions. Since, functional integration represents one of the essential stages of the brain processing, the extent of functional integration should be directly proportional to the brain ability to process information.
- The human brain network is the product of an intricate evolutionary optimization process that is largely unknown. Recent works has shown that human brain networks have a natural propensity to fault tolerance. Hence, node or edge ablation produces small effects if the cutting is limited and distributed. However, other evidences show that in many cases, structural damages of the brain network produce cascades of modifications in order to compensate the incoming failures, a phenomenon also known as *diaschis*.
- Physical connections are more reliable than functional ones that rely on strong statistical assumptions. In addition, scanning modalities for structural connections are more spatially accurate and reproducible. Many recent efforts were focused on the structural-to-functional relationship of brain networks. Among the proposed theories and methodologies, the network centrality appears a good candidate for the structural-to-functional network mapping. Namely, given a set of physical connections among nodes, the node and edge centralities are able to predict the load of node and edges within the information processing flow.

As a first step, we evaluated a small set of network centrality measures divided into two classes: the first one estimates node centrality through local network features (e.g., the node degree of the neighbor nodes), the second one approximates node centrality by using information of the entire network. Among the local centrality measures we preferred the node degree centrality, a quantitative measure of the number of node connections, whereas, for the measures of global centrality, we chose the betweenness (BC) and the eigenvector centralities (EC). The former measures the number of times a node is bridging neighboring or far nodes. The latter assigns a greater centrality to a preeminent node (a richly connected node) than to a poorly connected one.

**Figure 2:**
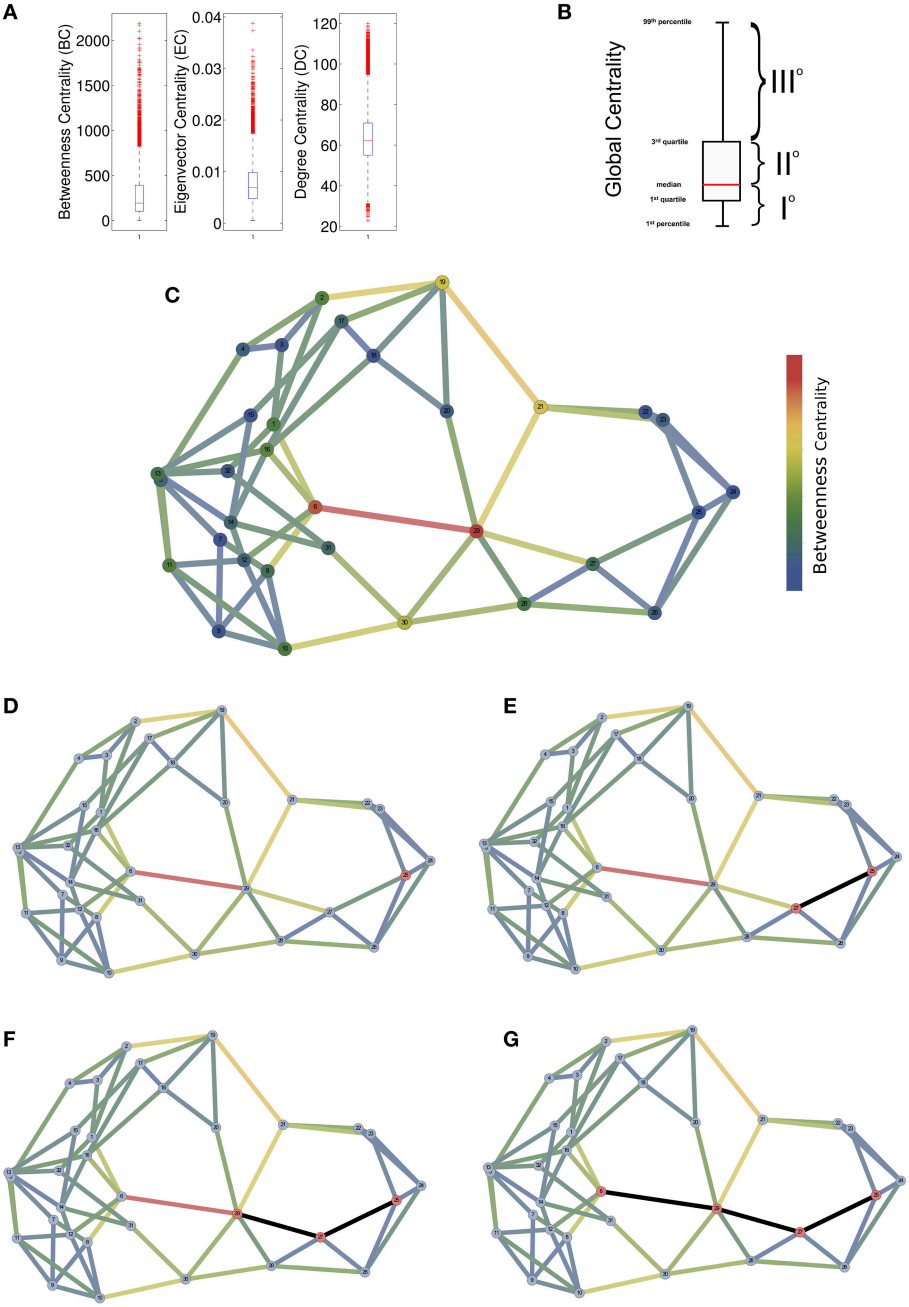
**Empirical explanation of the Compression Flow (CF) architecture**. **(A)** A comparison of the distributions of global centrality measures (Betweenness BC or Edge Betweenness EBC) vs. a local measure of centrality (Degree DC). Box-Whisker plots highlight the different nature of the metrics because the BC and the EBC present a heavy-tailed distribution while the DC shows a Gaussian-like distribution. **(B)** By generalizing from reports in **(A)**, global centrality measures appear to tripartite the node set by assigning to a first node subset small values of centrality (network periphery), to a second node subset large centrality values (network middle) and to a last node subset very large centrality values (network core). **(C)** A toy network with 32 nodes where edge and node colors indicate the centrality values. **(D)** Initially, the proposed algorithm randomly select a putative peripheral node (in red) and then it begins to walk by choosing the edge with a probability proportional to the edge weight. **(E–G)** Three steps of the random walk where selected nodes are colored with red and edges in black.

By analyzing the distribution of centrality in network topologies coherent with brain networks (see next section), we found that BC and EC showed heavy-tailed distributions (Figures 2A,B). The degree centrality on the contrary appeared normally distributed with a slight positive skewness (0.11). These results suggested that node loads were inhomogeneously distributed among nodes identifying groups of nodes that likely process a much higher amount of information than other ones.

For these reasons we assumed that betweenness centrality represents a predictor of the network information processing loads and we used it to predict the information flow from the periphery to the network core nodes. In this way, we created a direction for the random walks initiated from peripheral nodes and convergent to the network central nodes. The number of initially (and simultaneously) activated nodes corresponds to the input information size. Once activated, nodes prefer edges by randomly selecting one of its edges with a probability proportional to the edge weight (weighty edges have higher probability to be selected) and then the walk proceed to the inward node that became activate and the random selection starts again. In the random selection are excluded the inward edge that has activated the node itself in order to prevent loops along the random walk. Each walk ends when there are no more outward edges with a weight greater than the inward edge which indicates that further steps will get the walk out of the network center.

In more formal presentation, the following statements can introduce the algorithm:

Algorithm: Input: the adjacency matrix $M={\left([0,1]\subseteq \mathbb{R}\right)}^{n\times n}$ of the graph *G* = 〈*V, E*〉 with *v*_*i*_ ∈ {1, ⋯, *n*} and *E* = {(*i, j*)|*i, j* ∈ *V*}, the node betweenness centrality (BC) of *G*, the edge betweenness centrality (EBC) of *G*

Output: the extent of compression flow *CF* for the graph *G*

1. Set a pivot value ϑ in the BC distribution, usually a low percentile of the BC distribution (values from 5 to 10 do not affect results);
2. Establish which nodes have a BC lower than ϑ, thus obtaining the subset φ⊂*V* with |φ| = *k*of the putative most peripheral nodes of *G*;
3. For *t* = 1, ⋯, *k* compute and collect the random walks *r*_*t*_ from the periphery to the network center for each input load *t*; at each step the *t* activated nodes are randomly chosen from φ;
4. For *t* = 1, ⋯, *k* estimate the compression ratio by computing (through the *c* function) and counting the number of connected components |*c*(Ĝ)| of the graph provisional Ĝ obtained by the collection of all edges encountered in all paths of *r*_*t*_; the compression ratio is set to $\rho_{t}={\frac{t}{n-|c\left(Ĝ\right)|}}^{\prime };$
5. Sum up the obtained compression ratios $CF=\overset{k}{\underset{i=1}{\sum }}\rho_{i}$.

**Figure 3:**
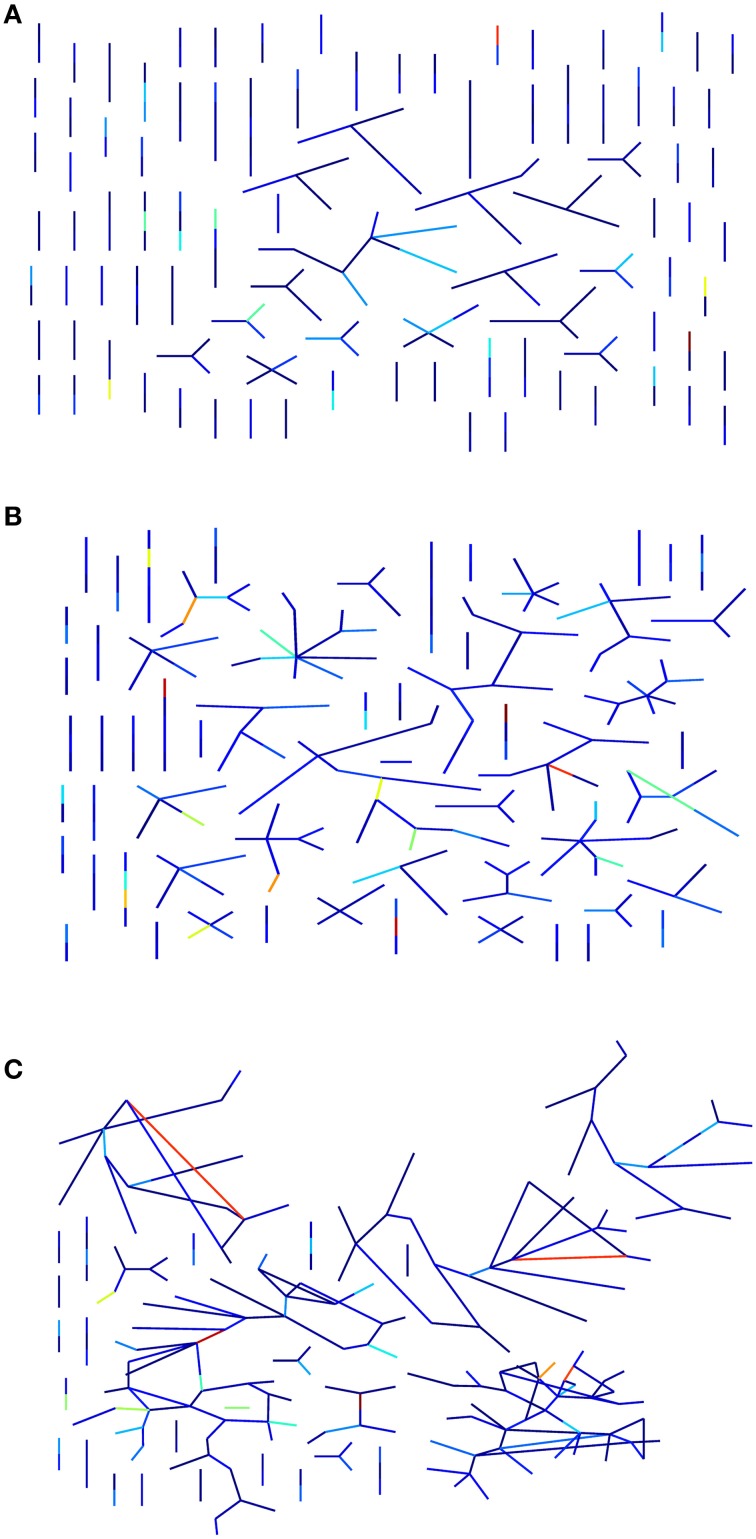
**Estimation of the information compression capability of a graph**. In the pictures, a graph layout that prefers edge visualization is adopted. In the final stage, the algorithm that estimates the CF, takes into consideration the random walks for each activation level (i.e., the number of randomly selected activated peripheral nodes). If there are many and small connected components then there are few convergent walks and so a poor compression capacity. **(A)** A network with a relatively small CF value is supported by the high number of connected component of the graph. Vice versa a newtwork with large CF has many less connected components **(C)**. **(B)** An intermediate case. Edge colors represent the edge betweenness centrality intensity.

The algorithm was written in Matlab and the code is publicly available at (https://sites.google.com/site/antoniogiulianozippo/codes). The rationale is illustrate by the toy example in Figures 2C–G and 3.

### Synthetic network models

Within the set of assumptions that brought us to develop the proposed method, we declared the working hypothesis that is the extent of what is ontologically defined as “functional information integration” could not be captured by the characteristic path length. Therefore, to validate the efficacy of the proposed method, we exert the CF and the L against two classes of network models. The first contained two brain-like topologies and the second contained two null model networks. Specifically, the former included the Watts-Strogatz model (WS) and the Barabasi-Albert model (BA) to generate respectively small-world and core-periphery networks (Senden et al., 2014). The latter included the Erdos-Renyi (ER) and the ring-lattice (RL) models to generate totally random and purely deterministic networks representing the null hypotheses.

## Results

In this work we aimed to propose a biomarker, the CF, of the AD able to quantify the severity of the impairment at different stages: early and late mild cognitive impairment (MCI, respectively EMCI and LMCI). To this purpose we considered the ADNI2 population endowed by functional modalities of neuroscanning, in particular we were interested in the 18FDG-PET and the resting-state fMRI. We selected functional volumes because one of the fundamental assumptions of the proposed method was that functional modifications of the whole-brain network occur before they appear in their structural imaging counterparts. Furthermore, with our approach we computed the whole-brain voxel-wise functional connectomes because we sustained that structural damages of the brain network produce non-local modifications even observable in apparently unrelated brain areas, an old theory named *diaschisis* proposed one century ago that has recently found many experimental supports (von Monakow, 1914; Carrera and Tononi, 2014; Fornito et al., 2015).

One of the fundamental dynamics of the human brain information processing is the functional integration. According to this theory the brain is able to collect and integrate information from many districts achieving representations of high order concepts and abstractions. As a strictly necessary effect, information undergoes to a sort of *lossy* data compression.

Consistently, the rationale of the proposed method is that since the human brain acts as proficient information compression device as expression of the fundamental ability to integrate information, altered connections due to amyloid accumulation should affect the compression capability. Starting from this assumption we estimated, for each functional connectome, the number of information compressing paths by means of a Monte Carlo-like approximation. With further steps, we subsequently obtained the CF, an estimation of the compression capability of the analyzed connectome.

We selected within the ADNI2 dataset, subjects that satisfied a cohort of conditions (see Materials and Methods) classified according to the initial clinical screening in one of the four classes: control (CR), early MCI (EMCI), late MCI (LMCI), and Alzheimer's disease (AD). The extracted whole brain voxel-wise connectomes were characterized both by a set of complex network statistics, namely the characteristic path length (L), the clustering coefficient (C), the modularity (Q), the node strength distribution (Ω), the number of network nodes (#Nodes), the node (BC), and the edge (EBC) betweenness centralities and by the proposed measure CF (see Table 2).

**Table 2:** **The complex network statistics used in this work**.

**Measure**	**Definition**	**Interpretation**
Node strength	$\Omega_{i}=\sum_{j\in N}a_{ij}$	Number of edges connected to a given node *i*. Nodes with relatively high values of *k* are called *hubs*
Shortest path length	$d_{ij}=\;\sum_{a_{fg}\;\in \;r_{i↔j}}1/a_{fg}$ where *r*_*i*↔*j*_ is the shortest path between *i* and *j*	The number of edges encountered in the shortest path between node *i* and *j*
Characteristic path length	$L=\;\frac{1}{n}\sum_{i\in N}L_{i}=\frac{1}{n}\sum_{i\in N}\frac{\sum_{j\in N,j\neq i}d_{ij}}{n-1}$	Measure of network integration
Clustering coefficient	$C=\;\frac{1}{n}\sum_{i\in N}C_{i}=\frac{1}{n}\sum_{i\in N}\frac{2t_{i}}{k_{i}(k_{i}-1)}$, with $t_{i}=\frac{1}{2}\sum_{j,h\in N}\sqrt[{3}]{a_{ij}a_{ih}a_{jh}}$	Measure of fine-grain network segregation. It counts the average number of triangles *t* (three-node fully connected graphs) present in the network
Modularity	$Q=\frac{1}{l}\sum_{u,v\in N}\left[a_{uv}-\frac{\Omega_{i}\Omega_{j}}{l}\right]\delta_{m_{i}}\delta_{m_{j}}$, where *l* is the sum of all weights of *V* (whose elements are called *modules*) and *m*_*i*_ is the module containing the node *i* and δ_*m*_*i*__ δ _*m*_*j*__ = 1 if *m*_*i*_ = *m*_*j*_ and 0 otherwise.	It evaluates the tendency of the network to be reduced in independent (or scarcely dependent) modules
Betweenness Centrality	$BC_{i}=\frac{1}{\left(n-1)\;(n-2\right)}\sum_{h,j\in N,h\neq j,h\neq i,i\neq j}\frac{\rho_{hj}(i)}{\rho_{hj}}$, where ρ_*hj*_ is the number of shortest paths between h and j, and ρ_*hj*_(*i*) is the number of shortest paths between *h* and *j* that pass through *i*	It is the amount of shortest paths that pass through the node *i*. It roughly indicates how much information burdens the node *i*

*We reported the weighted versions of each statistics. Weights are assumed to span from 0 to 1*.

### Validation on simulated network

Prior to apply our method on the selected ADNI2 population, we validated its goodness in two kinds of synthetic brain-like networks which underwent to incremental edge removal, a rough simulation of the brain tissue degeneration produced by amyloid accumulation. Specifically, we produced two groups of network models, one that contains two network models consistent with brain topologies and the other one contains two null networks as hypotheses to be rejected. Specifically, we considered the Watts-Strogatz model (WS) and the Barabasi-Albert model (BA) to generate respectively small-world and core-periphery networks (Senden et al., 2014). On the other hand, we examined Erdos-Renyi (ER) and the ring-lattice (RL) models to generate totally random and purely deterministic networks representing the null hypotheses. All network models had 2^10^ nodes (a size comparable to the observed connectomes) and the results of each model were averaged on 1000 runs to reduce the effects of randomness. We eventually compared results obtained with the CF measure with the classical statistics of functional integration L (the characteristic path length).

**Figure 4:**
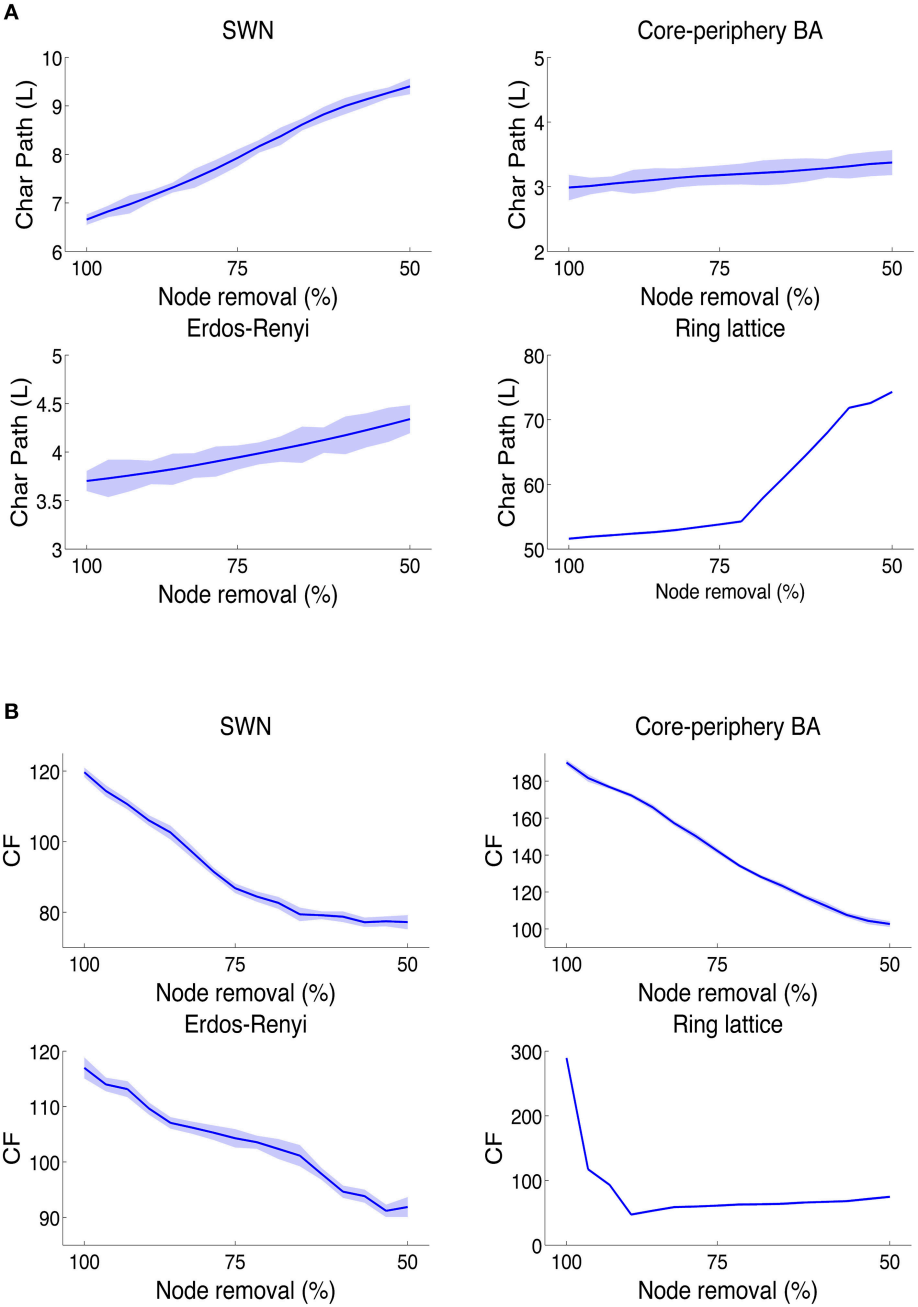
**Behavior of CF and L on synthetic networks**. In networks with brain-like topologies, the L values (that is inversely proportional to the information integration) **(A)** falls down, as effect of the random node removal, only in small-world network and not in the core-periphery Barabasi networks. **(B)** While by using the CF index, brain-like topologies abundantly lose values as effect of random node removals. Furthermore, this result also shows how brain-like networks became more and more inefficient to integrate information as the connections among nodes are lost as in diverse neurodegenerative diseases.

We found that L statistically detected deteriorations of network information integration capabilities in the WS (*P* = 0.000, *N* = 1000, Kruskal-Wallis test) model of brain network (Figure 4A) and in the RL (*P* = 0.000, *N* = 1000, Kruskal-Wallis test) and ER (*P* = 0.000, *N* = 1000, Kruskal-Wallis test) null models. However, L was unable to recognize the decline of information integration in the BA brain-like network (*P* = 0.326, *N* = 1000, Kruskal-Wallis test). Importantly, by using L we did not observe any statistical significance in the slight grow of L even when half of nodes were lost. This result indicated that the classical measure of information integration can be inadequate to disclose a fundamental dynamical feature in the brain network information processing.

Instead, we found that in WS and BA networks (Figure 4B), the CF fell down during edge removals (WS: *P* = 0.000, *N* = 1000, Kruskal-Wallis test; BA: *P* = 0.000, *N* = 1000, Kruskal-Wallis test), an effect more remarkable in BA than in WS. In fact, by removing half of nodes, WS networks lost 34% of compression capabilities whilst BA lost 46% of CF. Collaterally we observed also that CF was much higher in BA networks (+87%, *P* = 0.000, *N* = 1000, non-parametric Wilcoxon ranksum test) than in WS. In addition, we found that ER models reported a slower downfall of CF (-22%, *P* = 0.000, *N* = 1000, Kruskal-Wallis test). Eventually, the node removal altered the CF in RL networks (*P* = 0.000, *N* = 1000; Kruskal-Wallis tests) even when we randomly dropped only 6–7% of nodes, the CF decreased of almost 67% indicating that RL networks are scarcely tolerant to faults.

These results confirmed that on toy models of the human brain network the CF is able to detect the decline of information processing, a fact that did not always emerge by using the standard measure L.

### 18FDG-PET

By analyzing the complex network statistics, we concluded that measures such as C, L, Q, and the node strength were only able to discriminate AD group from CR group, but they misclassified EMCI and LMCI subjects into the CR groups. Specifically, the average clustering coefficient (C) was significantly higher in the CR (Tables 3, 4, ranksum tests with Bonferroni correction), EMCI, and LMCI groups than in the AD group whilst C did not differ between CR and EMCI, CR, and LCMI or between EMCI and LMCI. A similar result has been observed for Q while we found an opposite trend for L and Ω but the same discernibility of classes. Interestingly, the EBC and the BC were able to better separate groups by discriminating CR from LMCI subjects but failing to discern CR from EMCI patients. A similar behavior was found for the number of nodes (#Nodes) which discerned CR and AD but not EMCI from LMCI showing however a gradual decrease of the gray matter voxels. Remarkably, CF progressively decreased from CR to AD, significantly discriminate each class of the ADNI repository. These results revealed that the CF measure served as a biomarker of the dementia progression for the selected ADNI2 population in the three stages of the disease progression, from EMCI to AD.

**Table 3:** **Average values (and standard deviations) of the complex network statistics for the 18FDG-PET volumes**.

	**C**	**L**	**Q**	**Ω**	**BC**	**EBC**	**CF**	**#Nodes**
CR	0.450 ± 0.023	2.582 ± 0.188	0.333 ± 0.017	0.119 ± 0.012	4766 ± 377	2.260 ± 0.525	433 ± 23	3718 ± 193
EMCI	0.454 ± 0.025	2.579 ± 0.189	0.332 ± 0.017	0.123 ± 0.013	4468 ± 362	2.255 ± 0.518	405 ± 24	3456 ± 184
LMCI	0.459 ± 0.022	2.579 ± 0.181	0.334 ± 0.017	0.123 ± 0.013	3955 ± 336	2.170 ± 0.502	394 ± 21	3458 ± 187
AD	0.409 ± 0.021	2.730 ± 0.164	0.379 ± 0.020	0.071 ± 0.008	5291 ± 405	2.588 ± 0.556	377 ± 20	3191 ± 161

*C, indicates the clustering coefficient; L, the characteristic path length; Q, the modularity; Ω, the average node strength; BC, the betweenness centrality; EBC, the edge betweenness centrality; CF, the proposed measure called Compression Flow; and #Nodes represents the number of nodes*.

**Table 4:** ***P*-values of all the pairwise comparison of the classes (rows) of Table 3**.

	**C**				**L**				**Q**				**Ω**			
	**CR**	**EMC**	**LMC**	**AD**	**CR**	**EMC**	**LMC**	**AD**	**CR**	**EMC**	**LMC**	**AD**	**CR**	**EMC**	**LMC**	**AD**
CR	1	0.877	0.832	**0.012**	1	0.894	0.711	**0.000**	1	0.972	0.986	**0.003**	1	0.909	0.837	**0.000**
EMCI	0.877	1	0.978	**0.007**	0.894	1	0.995	**0.000**	0.972	1	0.998	**0.001**	0.909	1	0.999	**0.001**
LCMI	0.832	0.978	1	**0.000**	0.711	0.995	1	**0.000**	0.986	0.998	1	**0.000**	0.837	0.999	1	**0.000**
AD	**0.012**	**0.007**	**0.000**	1	**0.000**	**0.000**	**0.000**	1	**0.003**	**0.001**	**0.000**	1	**0.000**	**0.001**	**0.000**	1
	**BC**				**EBC**				**CF**				**#Nodes**			
CR	1	0.328	**0.010**	**0.012**	1	0.626	**0.011**	**0.000**	1	**0.003**	**0.012**	**0.001**	1	**0.000**	**0.009**	**0.000**
EMCI	0.328	1	**0.006**	**0.007**	0.626	1	**0.005**	**0.000**	**0.003**	1	**0.004**	**0.001**	**0.000**	1	0.792	**0.001**
LCMI	**0.010**	**0.006**	1	**0.000**	**0.011**	**0.005**	1	**0.000**	**0.012**	**0.004**	1	**0.000**	**0.009**	0.792	1	**0.000**
AD	**0.012**	**0.007**	**0.000**	1	**0.000**	**0.000**	**0.000**	1	**0.001**	**0.001**	**0.000**	1	**0.000**	**0.001**	**0.000**	1

*The non-parametric Wilcoxon ranksum test with Bonferroni correction was used in all comparisons (bold ones are significant). C, indicates the clustering coefficient; L, the characteristic path length; Q, the modularity; Ω, the average node strength; BC, the betweenness centrality; EBC, the edge betweenness centrality; CF, the proposed measure called Compression Flow; and #Nodes represents the number of nodes*.

**Figure 5:**
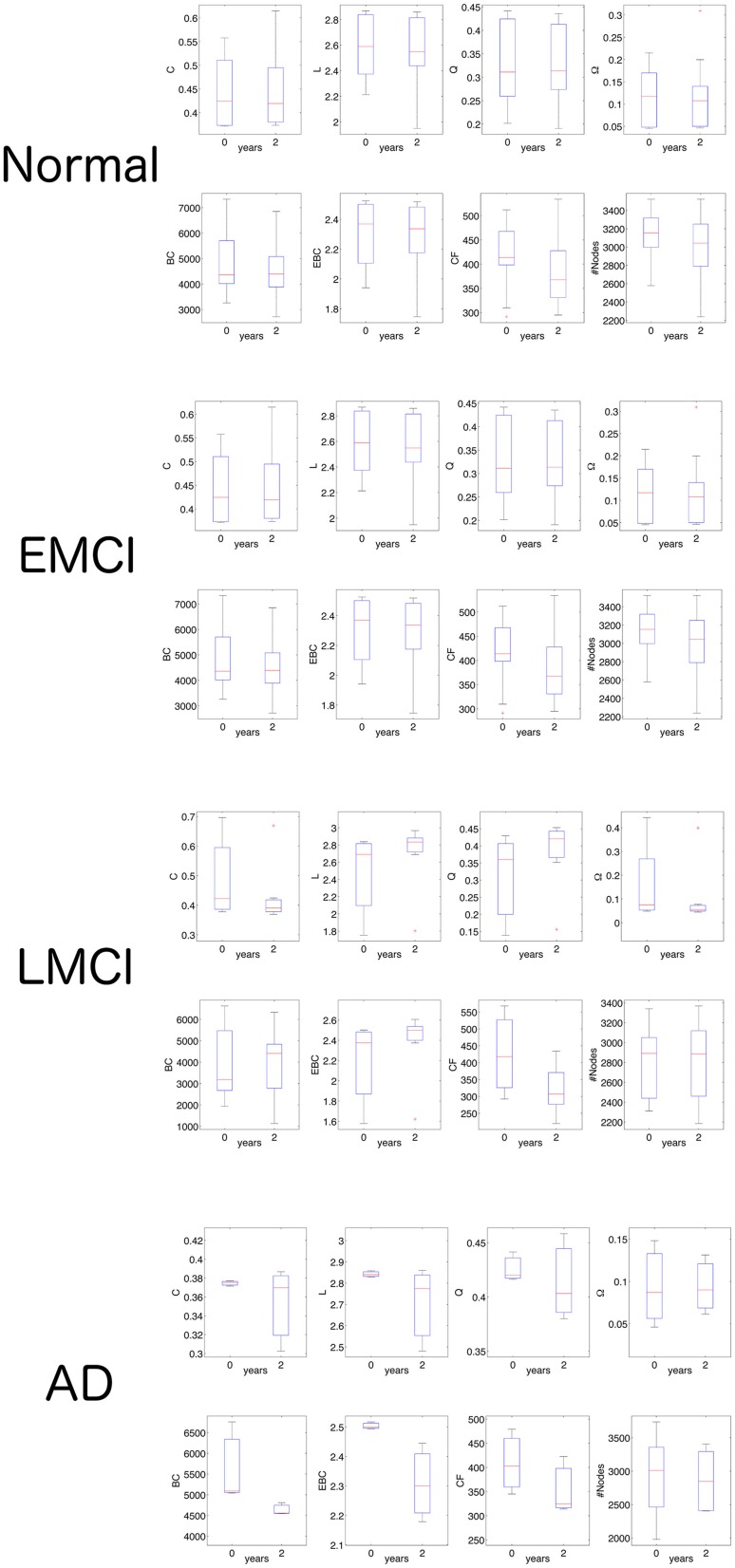
**Network statistics in the follow-up analysis of 18FDG-PET volumes**. The ranksum test shows that only CF is able to distinguish the two times of investigation (initial screening and after 2 years) in all three pathological classes (EMCI, LMCI, AD). For detailed significance see the Results section. C, indicates the clustering coefficient; L, the characteristic path length; Q, the modularity; Ω, the average node strength; BC, the betweenness centrality; EBC, the edge betweenness centrality; CF, the proposed measure called Compression Flow; and #Nodes represents the number of nodes.

Once established that CF was able to statistically separate each of the ADNI2 classes, we continued the investigation by analyzing CF values in the two times of scanning each class: at the initial screening and at after 2 years (Figure 5). In normal subjects we no found any statistical significance between network features at the two different times (non-parametric Wilcoxon ranksum test). In the group of EMCI patients, we observed significance for BC (*P* = 0.002, *N* = 18, ranksum test) and for CF (*P* = 0.000, *N* = 18, ranksum test). Namely, the betweenness centrality and the CF decreased in the 18FDG-PET connectomes acquired after 2 years of the initial screening. In the group of LMCI participants, we found again significance for CF (*P* = 0.000, *N* = 16, ranksum test) and for Q (*P* = 0.027, *N* = 16, ranksum test). Eventually the AD group revealed several significances: BC (*P* = 0.000, *N* = 17, ranksum test), EBC (*P* = 0.000, *N* = 17, ranksum test), CF (*P* = 0.001, *N* = 17, ranksum test) and #Nodes (*P* = 0.040, *N* = 17, ranksum test). Results of this section showed that CF was the only measure able to monotonically follow the impairment progression. Other measures like BC showed significance in EMCI and AD but not in LMCI.

### Resting-state fMRI

To assess the efficacy of the proposed biomarker CF to discriminate the dementia states, we repeated the same computational framework by replacing the 18FDG-PET functional volumes with the resting-state fMRI volumes (rs-fMRI).

Similarly, classical complex network statistics was able to distinguish CR from AD but failed to separate EMCI and LMCI from CR (C, L, Q, and Ω, Tables 5, 6). According to the previous results obtained with 18FDG-PET, BC, and EBC both added a finer characterization by discriminating CR from LMCI subjects but failing again to discern CR from EMCI patients.

**Table 5:** **Average values (and standard deviations) of the complex network statistics for the rs-fMRI volumes**.

	**C**	**L**	**Q**	**Ω**	**BC**	**EBC**	**CF**	**#Nodes**
CR	0.267 ± 0.013	3.237 ± 0.139	0.116 ± 0.009	0.240 ± 0.016	2951 ± 227	1.6939 ± 0.461	889 ± 42	4267 ± 217
EMCI	0.254 ± 0.011	3.327 ± 0.124	0.116 ± 0.008	0.238 ± 0.016	2930 ± 238	1.6954 ± 0.448	811 ± 37	4006 ± 202
LMCI	0.248 ± 0.010	3.389 ± 0.135	0.119 ± 0.011	0.230 ± 0.015	2629 ± 194	1.7428 ± 0.460	770 ± 28	3923 ± 189
AD	0.381 ± 0.037	2.794 ± 0.097	0.087 ± 0.008	0.358 ± 0.017	2643 ± 199	1.7502 ± 0.473	655 ± 24	3788 ± 170

*C, indicates the clustering coefficient; L, the characteristic path length; Q, the modularity; Ω, the average node strength; BC, the betweenness centrality; EBC, the edge betweenness centrality; CF, the proposed measure called Compression Flow; and #Nodes represents the number of nodes*.

**Table 6:** ***P*-values of all the pairwise comparison of the classes (rows) of Table 5**.

	**C**				**L**				**Q**				**Ω**			
	**CR**	**EMC**	**LMC**	**AD**	**CR**	**EMC**	**LMC**	**AD**	**CR**	**EMC**	**LMC**	**AD**	**CR**	**EMC**	**LMC**	**AD**
CR	1	0.877	0.832	**0.012**	1	0.894	0.711	**0.000**	1	0.972	0.986	**0.003**	1	0.909	0.837	**0.000**
EMCI	0.877	1	0.978	**0.007**	0.894	1	0.995	**0.000**	0.972	1	0.998	**0.001**	0.909	1	0.999	**0.001**
LCMI	0.832	0.978	1	**0.000**	0.711	0.995	1	**0.000**	0.986	0.998	1	**0.000**	0.837	0.999	1	**0.000**
AD	**0.012**	**0.007**	**0.000**	1	**0.000**	**0.000**	**0.000**	1	**0.003**	**0.001**	**0.000**	1	**0.000**	**0.001**	**0.000**	1
	**BC**				**EBC**				**CF**				**#Nodes**			
CR	1	0.328	**0.010**	**0.012**	1	0.626	**0.011**	**0.000**	1	**0.003**	**0.012**	**0.001**	1	**0.000**	**0.009**	**0.000**
EMCI	0.328	1	**0.006**	**0.007**	0.626	1	**0.005**	**0.000**	**0.003**	1	**0.004**	**0.001**	**0.000**	1	0.792	**0.001**
LCMI	**0.010**	**0.006**	1	**0.000**	**0.011**	**0.005**	1	**0.000**	**0.012**	**0.004**	1	**0.000**	**0.009**	0.792	1	**0.000**
AD	**0.012**	**0.007**	**0.000**	1	**0.000**	**0.000**	**0.000**	1	**0.001**	**0.001**	**0.000**	1	**0.000**	**0.001**	**0.000**	1

*The non-parametric Wilcoxon ranksum test with Bonferroni correction was used in all comparisons (bold ones are significant). C, indicates the clustering coefficient; L, the characteristic path length; Q, the modularity; Ω, the average node strength; BC, the betweenness centrality; EBC, the edge betweenness centrality; CF the proposed measure called Compression Flow; and #Nodes represents the number of nodes*.

By using rs-fMRI, CF progressively and significantly decreased in each analyzed conditions (CR, EMCI, LMCI, AD, respectively) confirming that CF can represent a proficient biomarker of the AD progression. In addition, we compared the significance levels obtained from 18FDG-PET and rs-fMRI analyses and we concluded that rs-fMRI was more accurate than 18FDG-PET with each network statistics.

**Figure 6:**
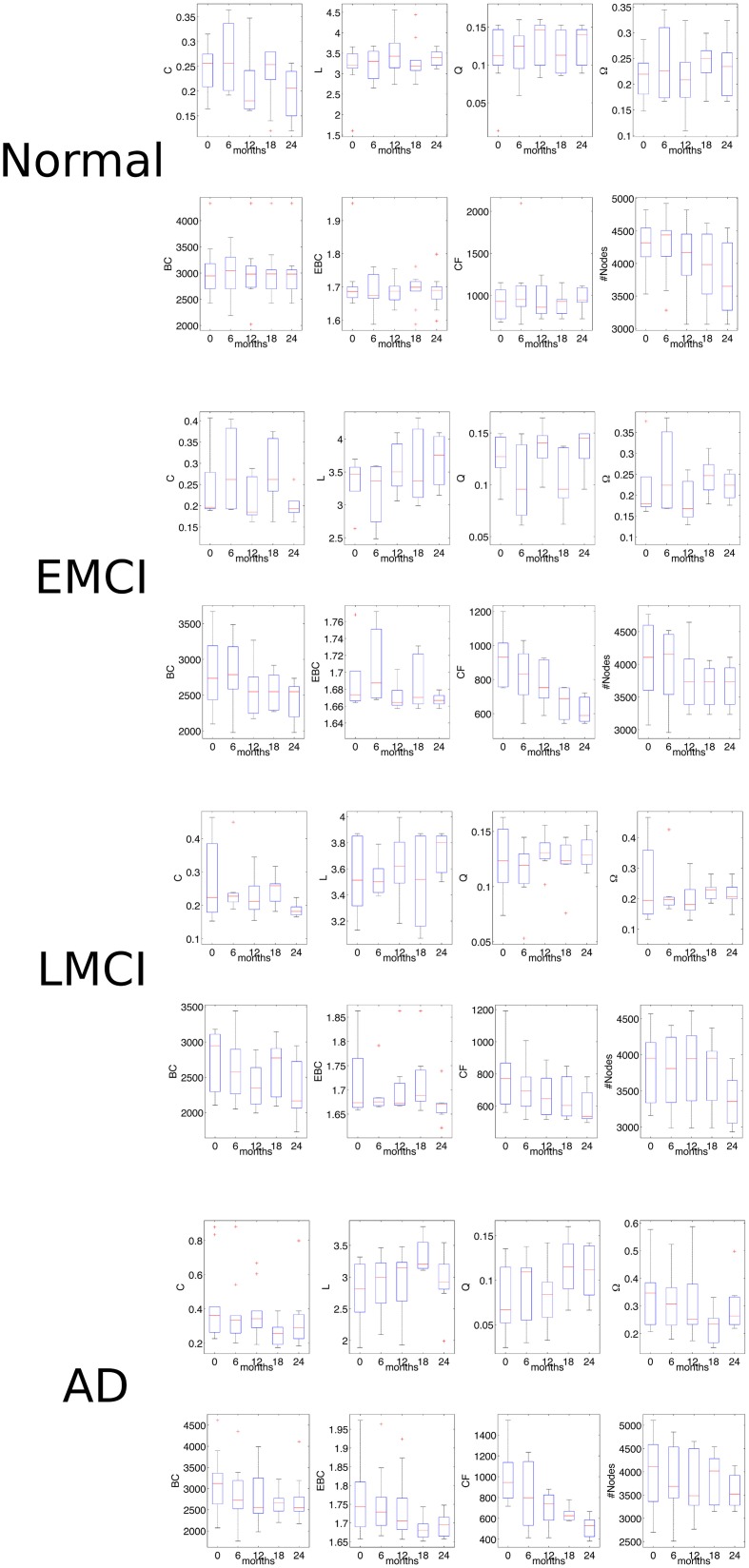
**Network statistics in the follow-up analysis of rs-fMRI volumes**. The Mann-Kendal monotonic trend test shows that only CF is able to distinguish the two times of investigation (initial screening and after 2 years) in all three pathological classes (EMCI, LMCI, AD). For detailed significance see the Results section. C, indicates the clustering coefficient; L, the characteristic path length; Q, the modularity; Ω, the average node strength; BC, the betweenness centrality; EBC, the edge betweenness centrality; CF, the proposed measure called Compression Flow; and #Nodes represents the number of nodes.

We further wondered whether CF was able to followed the impairment progression at the five times available in the selected ADNI2 subset: at initial screening, after 6 months, after 12 months, after 18 months, and after 24 months (Figure 6). In normal subjects we no found any statistical significance between network features at the five different times (non-parametric Mann-Kendal monotonic trend test) although CF appeared to monotonically decrease after the 6 months (*P* = 0.009, *N* = 24, Mann-Kendal test). In the group of EMCI patients, we observed significance for CF (*P* = 0.000, *N* = 18, ranksum test). Namely, the CF progressively decreased in the rs-fMRI connectomes acquired along the 2 years of the initial screening. In the group of LMCI participants, we also found significance for CF (*P* = 0.000, *N* = 16, ranksum test). Eventually the AD group revealed further significance only for CF (*P* = 0.000, *N* = 17, ranksum test). These last results indicated that CF was the only measure able to monotonically follow the impairment progression.

## Discussions

In this work we surveyed data from a database, the ADNI repository (version 2), and analyzed them in the light of a novel measure, the CF by which a finely detailed estimate of large brain network functional integration is attained. On the voxelwise 18FDG-PET and resting-state fMRI (rs-fMRI) connectomes, the CF, diversely from many common complex network statistics, was able to statistically discriminate each of the considered clinical states (normal CR, early mild cognitive impairment EMCI, late mild cognitive impairment LMCI, and Alzheimer's Disease AD). Furthermore, CF was also able to characterize the progression of the disease within each pathological class (EMCI, LMCI, and AD) finely following the extent of the impairment. To the best of our knowledge, this measure can be considered as a promising AD non-invasive biomarker.

### The neurophysiological inheritance of AD

Functional imaging from fMRI and 18FDG-PET acquisitions were related onto the structural MRI counterparts. The study had the aim to identify hidden or previously undetected signs of functional degeneration still unexpressed at the clinical phenomenological level in AD or MCI patients. We selected strongly restrictive statistical requirements in order to reduce the “noise” introduced necessarily by large data arrays. The CF measure allowed for discriminating EMCI, LMCI, AD, and normal subjects just starting from the sole brain connectomics. More importantly, MCI and full expressed ADs were similarly distinguishable, a previously unattained estimate with heavy prognostic and therapeutic value. These results have special interest. As first it is assumable that the abstract space of prodromal neurodegenerative signs, incorporating a multiform semiological palette of emblems of cognitive degeneration, is richer than thought of different, and clinically relevant, hues. Additionally, this aggregate aside those subsets expressing conditions naturally evolving to AD, yet shows others with distinguishable structures and different outcomes from AD, all factors that deserve to be further investigated. It goes no saying that these neurodynamical signs must be matched with finer clinical investigations and with biochemical data but they can represent a step forward to a more robust prognostic and therapeutic strategy.

The measure has a strong dynamic flavor taking into consideration the information workflow both in terms of global network centrality. The procedure itself for measuring CF asks for an arbitrary setting of a pivot in a node within the betweenness centrality distribution with the observation of a centrality-driven development of a random walk, a method that doesn't represent a necessary or preliminary privilege of some already chosen trajectory. This strategy allows a more natural estimate of implicit functional properties of the network dynamics.

Cautions must be taken into consideration. While fMRI measures are implicitly related on actual local measures of BOLD signal (a measure that delivers the ratio between hemoglobin oxygenation levels), images from FDG-PET measures are weighted over a mean estimate of glucose consumption, an assumption that doesn't meet the actual finding that different cerebral regions may display very diverse blood vessel architectures and related dynamics (Zippo and Castiglioni, 2015). This implies an implicit misalignment between fMRI and FDG-PET data. However, due to the still missing fine grained observation at the dynamic level, the imbalance can be still tolerated. The prognostic value of the proposed technique instead deserves a deeper appraisal. It could become one of the key points to decide for a therapeutic choice. In the future, the use (or less) of drugs relenting the development of a full-blown AD syndrome or disease could be decided on the preliminary diagnostic appraisal well partitioning the space of AD/nonAD neurodegeneration dominions.

Besides the clinical involvements of the CF as neuroimaging biomarker, the fundamental assumptions on which the method is based could shed new insights in the comprehension of AD (Rinaldi et al., 2015). The integration of information represents one of the most important information processing stage of the brain and aberrations of information integration are reported in a plethora of diseases. Because CF is an alternative estimator of the information integration, we have previously concluded that AD and its prodromal stages are significant marked by a strong impairment of information integration. Although information integration is unlikely the cause of the AD progression but rather one of the many halfway consequences, the mere restoration through pharmacological of electrical intervention could temporarily and partially restore information transmission efficiency toward physiological levels. Indeed, recently, a latent feature of antidepressants have been found by Schaefer et al. (2014) which reported that antidepressants enhance and promote the functional integration of the brain, a short-term effect that eventually boost the neurogenesis and the white matter development. Apparently in accordance with this speculation another recent study of Sheline and collegues showed that a specific antidepressant molecule (citalopram) decreases the production of CSF amyloid beta (Aβ) in humans (Sheline et al., 2014).

### Past works

The analysis of brain networks with complex network statistics is field largely debated. Wang and colleagues recently showed that AD patients can be easily distinguished from normal subjects by network properties extracted from electroencephalographic functional connectomes (Wang et al., 2014). Other authors found that functional connectivity strength of AD are reduced and a weaker correlation with MCI patients can be observed (Zhou et al., 2015). Furthermore, the classical information integration statistics L has been observed to be worsen in AD patients while the clustering coefficient C resulted invariant (Stam et al., 2007). An apparently contradictory results because C and L are usually correlated in brain-like topologies (Wang et al., 2007; Catricalà et al., 2015). From a more purely topological perspective, a recent work indicates that the only measure of small-worldness encloses the discriminability power between normal and AD subjects (Supekar et al., 2008). More specifically, other authors found that AD can be the result of the removal of important nodes called hubs (nodes with higher degree that typically have also higher centrality; Buckner et al., 2009; Sheline and Raichle, 2013; Brier et al., 2014a,b).

## Limitations and conclusions

There is a latent necessity in the proposed approach to consider only large-scale networks. The method of CF better approximates the compression-based functional integration through random centrality-driven walks and for network of less than one hundred nodes the estimation results meaningless. This drawback represents a clear drawback of the approach and also justifies the choice to use voxelwise connectomes instead of ROI-based which are limited to few hundreds of parceled regions. The computation of the edge and node betweenness centrality could represent a consequential limitation because the algorithm leads to a cubic computational complexity slowing down the analyses of large sets of volumes. Nonetheless, promising suboptimal algorithms has been recently introduced by enhancing the computational times also with the aid of parallel computing architectures (Geisberger et al., 2008; Bonneau et al., 2009; Chan et al., 2009; Kourtellis et al., 2013).

In conclusion this work claims that a novel measure of information integration based on an intuitive approximation of the network compression capabilities through random centrality-driven walks is able to fully characterize both the single CR, EMCI, MCI, and AD classes and the times of disease progression in 18FDG-PET and rs-fMRI volumes.

Eventually, since the synthesis of the CF method has been driven by several fundamental theoretical tenets, it could be used in other neurological, psychiatric and psychological conditions as well as in fundamental neuroscientific studies about the information integration in the human brain networks.

### Conflict of interest statement

The authors declare that the research was conducted in the absence of any commercial or financial relationships that could be construed as a potential conflict of interest.
